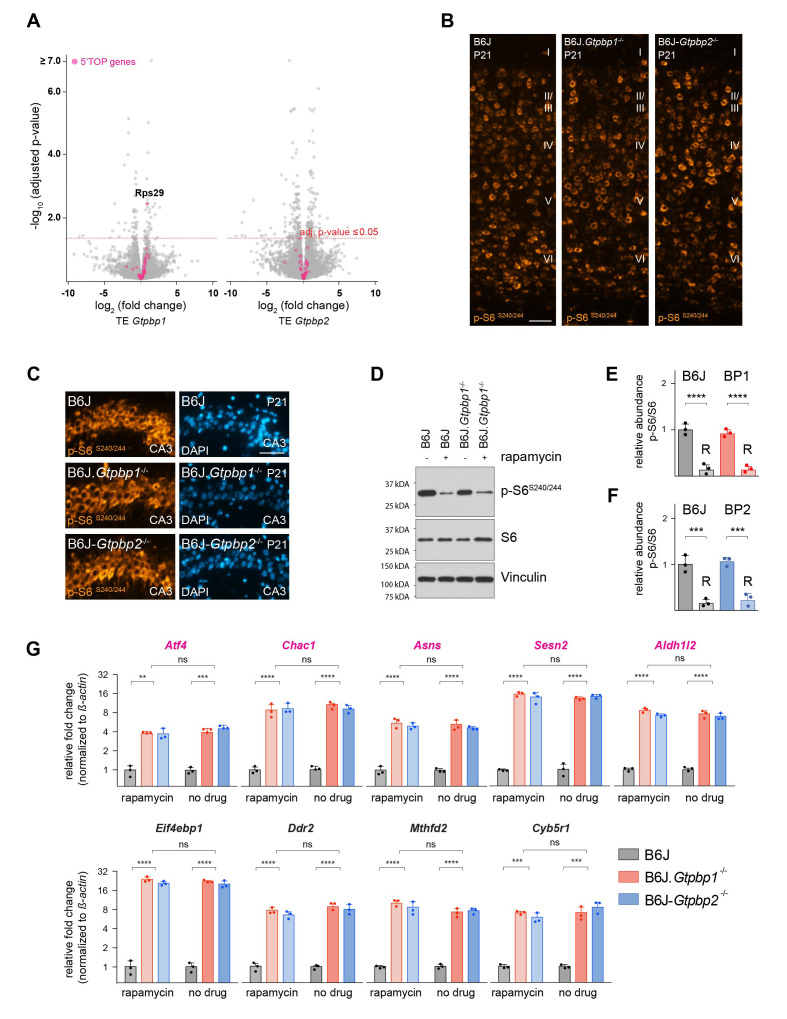# Correction: GTPBP1 resolves paused ribosomes to maintain neuronal homeostasis

**DOI:** 10.7554/eLife.70785

**Published:** 2021-06-04

**Authors:** Markus Terrey, Scott I Adamson, Alana L Gibson, Tianda Deng, Ryuta Ishimura, Jeffrey H Chuang, Susan L Ackerman

Terrey M, Adamson SI, Gibson AL, Deng T, Ishimura R, Chuang JH, Ackerman SL. 2020. GTPBP1 resolves paused ribosomes to maintain neuronal homeostasis. *eLife* **9**:e62731. doi: 10.7554/eLife.62731.

Published 13, November 2020

We discovered that one of our biological replicates for our ribosome profiling experiments from B6J-Gtpbp2 ^-/-^ cerebellum was inadvertently duplicated during analysis. We have redone the analysis using the appropriate file, and the actual numbers of genes that have ribosome stalling or changes in translational efficiency in Fig 2C, Figure 2-figure supplement 1 Panel B and C, and Figure 4-figure supplement 1 Panel A are slightly changed. These figures have been updated to reflect this correction and the text related to Figure 2C has also been corrected. We have also updated the associated excel sheets- Supplementary file 1 (NMF205 pause genes) and Supplementary file 3 (TE NMF205). The data in GEO have been updated and the accession number remains the same. The article has been corrected accordingly and the corrections do not in any way change our conclusions.

In the Results:

“Only one of the 53 detected 5’TOP genes had an altered TE in the B6J.*Gtpbp1*^-/-^ cerebellum (*Rps29*, TE *Gtpbp1*) or in the B6J-*Gtpbp2^-^*^/-^ cerebellum (*Rplp0*, TE *Gtpbp2*) compared to the B6J cerebellum (*Supplementary file 3*, Figure 4-figure supplement 1A).

Replaces the following text:

“Only one (RPS29) of the 53 detected 5’TOP genes had an altered TE in the B6J.*Gtpbp1*^-/-^ cerebellum compared to the B6J cerebellum (TE *Gtpbp1, Supplementary file 3*) and none of the 5’TOP genes displayed TE alterations in the B6J-*Gtpbp2^-^*^/-^ cerebellum relative to the B6J cerebellum (TE *Gtpbp2, Supplementary file 3*) (Figure 4-figure supplement 1A).

The updated Figure 2 (panel B and C) is shown here:

**Figure 1:**
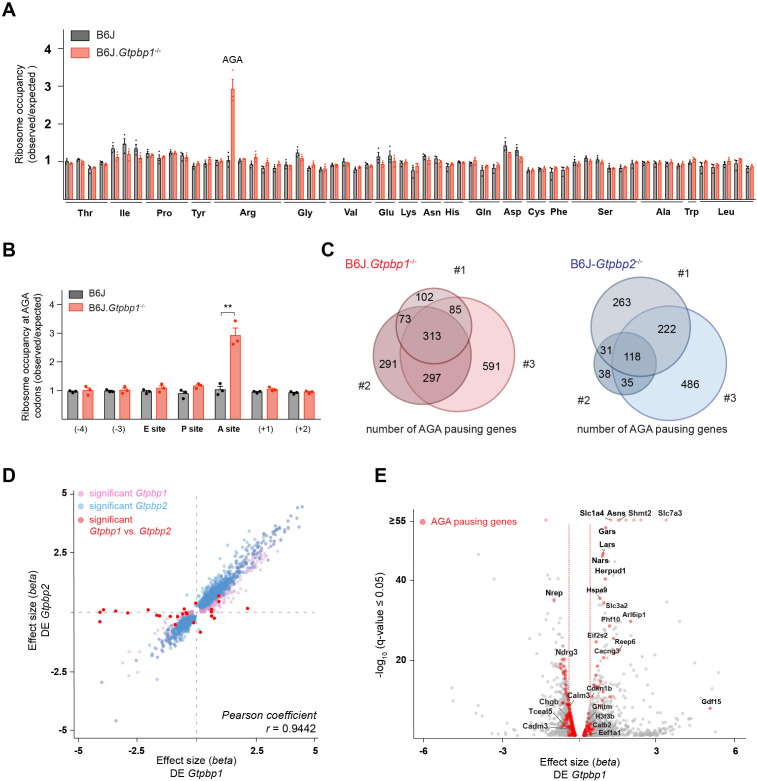


The originally published Figure 2 is also shown for reference:

**Figure 2:**
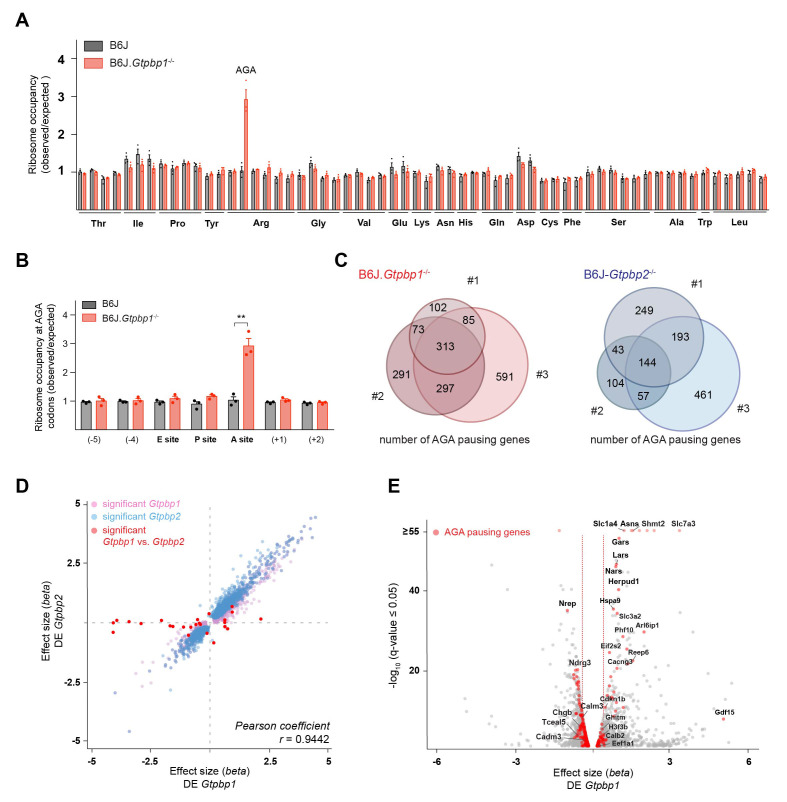


The updated Figure 2-figure supplement 1 (panel C) is shown here:

**Figure 3:**
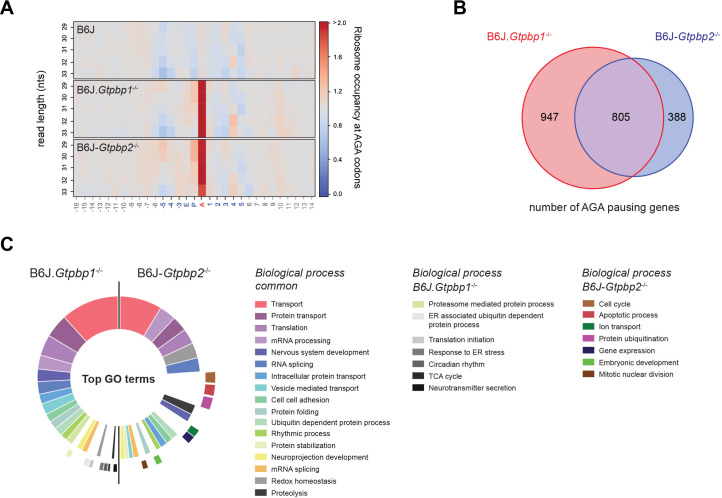


The originally published Figure 2-figure supplement 1 is also shown for reference:

**Figure 4:**
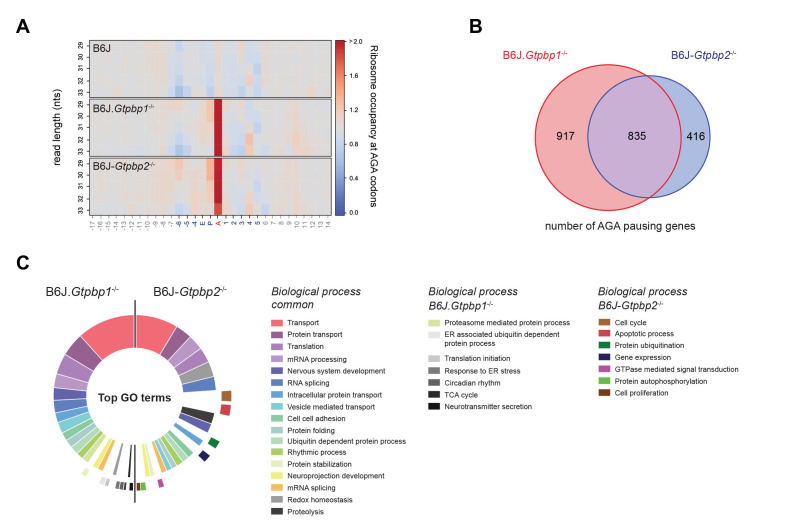


The updated Figure 4-figure supplement 1 (panel A) is shown here:

**Figure 5:**
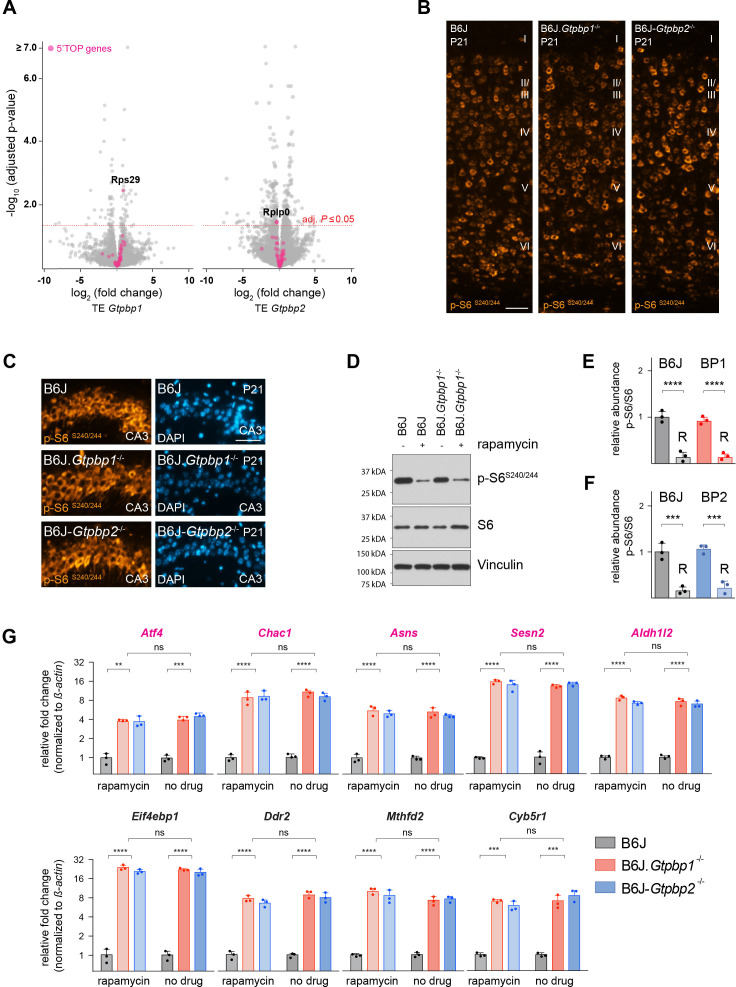


The originally published Figure 4-figure supplement 1 is also shown for reference:

**Figure 6:**